# Population Pharmacokinetics of Intravenous Acyclovir in Oncologic Pediatric Patients

**DOI:** 10.3389/fphar.2022.865871

**Published:** 2022-04-14

**Authors:** Natalia Maximova, Daniela Nisticò, Giacomo Luci, Roberto Simeone, Elisa Piscianz, Ludovica Segat, Egidio Barbi, Antonello Di Paolo

**Affiliations:** ^1^ Department of Pediatrics, Institute for Maternal and Child Health—IRCCS Burlo Garofolo, Trieste, Italy; ^2^ Department of Medical, Surgical and Health Sciences, University of Trieste, Trieste, Italy; ^3^ Department of Clinical and Experimental Medicine, University of Pisa, Pisa, Italy; ^4^ Transfusion Medicine Department, Azienda Sanitaria Universitaria “Giuliano Isontina”, Trieste, Italy; ^5^ Laboratory for Hygiene and Public Health, University Hospital of Trieste, Trieste, Italy

**Keywords:** acyclovir, pediatric patients, hematopoietic stem cell transplantation, pharmacokinetics, non-linear mixed effect modeling, prolonged infusion acyclovir, children, prolonged infusion

## Abstract

**Background:** Acyclovir represents the first-line prophylaxis and therapy for herpes virus infections. However, its pharmacokinetics in children exposes them to the risk of ineffective or toxic concentrations. The study was aimed at investigating the population pharmacokinetics (POP/PK) of intravenous (IV) acyclovir in oncologic children.

**Methods:** Patients (age, 8.6 ± 5.0 years, 73 males and 47 females) received IV acyclovir for prophylaxis (*n* = 94) and therapy (*n* = 26) under a therapeutic drug monitoring (i.e., minimum and maximal plasma concentrations, >0.5 and <25 mg/L, respectively). Plasma concentrations were fitted by nonlinear mixed effect modeling and a simulation of dosing regimens was performed. Findings were stratified according to an estimated glomerular filtration rate (eGFR) threshold of 250 ml/min/1.73 m^2^.

**Results:** The final 1-compartment POP/PK model showed that eGFR had a significant effect on drug clearance, while allometric body weight influenced both clearance and volume of distribution. The population clearance (14.0 ± 5.5 L/h) was consistent across occasions. Simulation of standard 1-h IV infusion showed that a 10-mg/kg dose every 6 h achieved target concentrations in children with normal eGFR (i.e., ≤250 ml/min/1.73 m^2^). Increased eGFR values required higher doses that led to an augmented risk of toxic peak concentrations. On the contrary, simulated prolonged (i.e., 2 and 3-h) or continuous IV infusions at lower doses increased the probability of target attainment while reducing the risk of toxicities.

**Conclusion:** Due to the variable pharmacokinetics of acyclovir, standard dosing regimens may not be effective in some patients. Prospective trials should confirm the therapeutic advantage of prolonged and continuous IV infusions

## Introduction

Herpesvirus infections in immunocompromised patients, particularly in hematopoietic stem cell transplant (HSCT) recipients, lead to severe disease with high dissemination rates, complications, and mortality (Beyar-Katz et al., 2020). Herpes simplex virus (HSV) is a ubiquitous virus that results in lifelong infections due to its ability to alternate between lytic replication and latency (Ly et al., 2021). The worldwide prevalence of HSV-1 increases consistently with age, reaching 40% by age 15 and increasing to 60%–90% in older adults (Chayavichitsilp et al., 2009). Up to 80% of adult leukemia patients are HSV seropositive, as well as up to 80% of HSV-seropositive allogeneic HSCT recipients had post-transplant HSV reactivation (Styczynski et al., 2009; Flowers et al., 2013). In the first post-transplant year, symptomatic varicella-zoster virus (VZV) reactivation in adult recipients is described with rates of 13%–55% (Beyar-Katz et al., 2020). Similarly, 30%–33% of pediatric HSCT recipients had VZV reactivation, and 11% of these were disseminated (Fisher et al., 2008). In the current era of antiviral prophylaxis in seropositive HSCT recipients, the infection rate has decreased significantly, besides a significant reduction in mortality (Dadwal, 2019).

In pediatric patients undergoing allogeneic HSCT, surveillance algorithms, antiviral prophylaxis, or pre-emptive treatment are well established for many viruses due to the high incidence of severe systemic complications in this population (Czyzewski et al., 2019; Jaing et al., 2019). In contrast to HSCT recipients, data on systemic viral infections in children receiving chemotherapy for hematological malignancies are very limited (Buus-Gehrig et al., 2020). However, few reports confirm that children with malignant diseases who experience prolonged periods of myelosuppression due to cytotoxic chemotherapy are highly susceptible to invasive viral infections (Feldman and Lott, 1987).

Most viral reactivation in adult cancer patients during neutropenia after myelotoxic chemotherapy is due to HSV (Saral et al., 1984). However, despite the high infection rate, there is not enough evidence from randomized trials on acyclovir prophylaxis in patients with acute leukemia to establish a strong recommendation in adult and pediatric patients undergoing intensive chemotherapy (Styczynski et al., 2009).

Acyclovir (ACV) effectively prevents and treats HSV and VZV infections but demonstrates high interindividual variability in its treatment response. Indeed, ACV prophylaxis is recommended for all HSV-seropositive HSCT recipients from conditioning until engraftment or until mucositis resolves to prevent HSV reactivation during the early post-transplant period. For VZV-seropositive HSCT recipients antiviral prophylaxis is recommended for at least one year, while for VZV-seronegative HSCT recipients passive immunization is preferred (Tomblyn et al., 2009; Carreras et al., 2018). For instance, the prophylactic regimen of ACV for the European Blood and Marrow Transplant Group consists in 250 mg/m^2^ (or 5 mg/kg) i.v., every 12 h, while the treatment of infections needs an intensified regimen (250 mg/m^2^ or 5 mg/kg i.v., every 8 h for 7–10 days) (Carreras et al., 2018). Due to the time-dependent killing of ACV, plasma concentrations should be higher than 1 mg/L for at least 50% of time interval between two consecutive doses (Saiag et al., 1999), so that a minimum plasma concentrations >0.5 mg/L could be considered an appropriate target. Moreover, high peak concentrations in plasma (i.e., >50 mg/L) are associated with an increased rick of neurotoxicity (Wade and Monk, 2015), even if the correlation between high plasma concentrations and the risk of nephrotoxicity and bone marrow adverse events remains to be fully investigated.

ACV is eliminated by the kidney’s glomerular filtration and tubular secretion, has low oral bioavailability, approximately 10% in children (Carcao et al., 1998), and a short half-life, hence it requires high and repeated doses to exceed the value of the inhibitory concentration (Abdalla et al., 2020). Furthermore, ACV has high interindividual variability (Blum et al., 1982; O'Brien and Campoli-Richards, 1989), which is particularly evident in the younger patients, and related to changes in renal function during the first months after the birth and body weight across the ages (De Miranda and Blum, 1983; Zeng et al., 2009; Abdalla et al., 2020). In addition to this, the genetic status of the patient may be considered a further cause of variability of pharmacokinetics and clinical outcome, as recently demonstrated for the NUDT15 polymorphism (Nishii et al., 2021).

Therefore, information regarding ACV optimal use in children with malignancies is restricted because pharmacokinetic data are limited in this population (Zeng et al., 2009; Abdalla et al., 2020). This study aims to characterize the pharmacokinetics of ACV following intravenous (IV) administration and to evaluate the adequacy of current dose regimens for children with malignancy undergoing myelosuppressive chemotherapy or HSCT. Additionally, the study aims to explore alternative dosing regimens that could be more effective and tolerable.

## Materials and Methods

### Study Design and Population

This prospective, single-center, observational study was carried out at the pediatric Onco-Hematology Department and pediatric Bone Marrow Transplant Center of the Institute for Maternal and Child Health—IRCCS “Burlo Garofolo,” Trieste, Italy, from 2011 to 2020. The Institutional Review Board of the IRCCS Burlo Garofolo (reference no. IRB RC 10/20) approved the protocol, and the study was conducted following the Declaration of Helsinki (Clinicaltrials.gov code: NCT05198570). The patients’ parents gave their written consent to collect and use personal data for research purposes. From January 2011 to December 2020, consecutive patients aged 0–18 years, affected by hematological malignancies, undergoing ACV prophylaxis or treatment for HSV-VZV infection during allogeneic HSCT or ACV treatment during high-intensity chemotherapy were included in this study. All patients underwent ACV therapeutic drug monitoring (TDM), and consequent dose adjustment was applied to maintain minimum (C_min_) and maximum (C_max_) plasma concentrations >0.5 mg/L and <25 mg/L, respectively, as per laboratory practice. Data collection included patient demographic characteristics as gender, age, weight, height, body mass index, body surface area, serum creatinine in addition to primary diagnosis, treatment for primary diagnosis, ACV dose, administration interval, concomitant medications, duration of ACV treatment, and cause of treatment interruption. The Schwartz formula determined the estimated glomerular filtration rate (eGFR) for each patient (Schwartz et al., 1976).

### ACV Administration Regimens and Blood Sampling

ACV was administered every 6–8 h intravenously (Acyclovir Recordati^®^, Biologici Italia Laboratories S.r.l., Masate, Milan, Italy) over 60 min infusion, with median (range) starting daily doses equal to 40.7 (15.6–136.7) mg/kg/day. The prescribed doses refer to the local protocols and depend on the age, treatment indication, and renal clearance. These differences in dosage reflect systematic changes in institutional practice over the ten years covered by the study. The first pharmacokinetic assessment (two samples) was performed after at least four days of ACV administration when the steady state was achieved. In particular, blood samples were withdrawn 10 min before (trough levels, C_min_) or 30 min after IV infusion (maximum concentration, C_max_). For some patients, blood samples were collected on several occasions. Blood samples were centrifuged, and plasma concentrations of ACV were measured by a liquid chromatography-tandem mass spectroscopy (LC-MS/MS) performed in multiple reaction monitoring mode (Kanneti et al., 2009). Briefly, after deproteinization, calibration standards, quality controls (both prepared in human blank plasma) and patients’ plasma samples (using fluconazole as internal standard, IS) were prepared by solid phase extraction (Oasis HLB Vac Cartridge, Waters, Milford, CT, United States). Acyclovir and IS were eluted through a C18 column with a mobile phase consisting of 0.1% formic acid solution and methanol (30:70 v/v) at a flow rate of 0.8 ml/min in isocratic conditions. The LC-MS apparatus worked in positive ion detection, and quantification was performed in multiple reactions monitoring (MRM) of transition ions m/z 226.3 > 152.1 and m/z 306.9 > 219.9 for ACV and IS, respectively.

### Pharmacokinetic Modeling and Simulation

The population pharmacokinetics (POP/PK) analysis was performed on the available plasma concentrations using a nonlinear mixed effect modeling approach using NONMEM software vers. 7.4 (ICON, Dublin, Ireland)^1^ and the packages PsN and Xpose (Jonsson and Karlsson, 1999; Lindbom et al., 2004). One- and two-compartment models with first-order elimination were tested, while the residual error was assayed as an additive, proportional and mixed model. Interoccasion variability (IOV) was evaluated for pharmacokinetic parameters. The introduction of covariates within the model was guided by their range of values in the dataset and their possible mechanistic involvement in ACV pharmacokinetics. Overall, a decrease in the objective function value (OFV) greater than 3.84 points (*p* < 0.05) and 6.63 points (*p* < 0.01) in the forward inclusion and backward exclusion, respectively, were considered during the model development. The conditional weighted residuals (CWRES) were calculated (Hooker et al., 2007), and the goodness-of-fit (GOF) plots, the precision of parameter estimates, η- and ε-shrinkage values were evaluated for each model. A bootstrap analysis and a prediction-corrected visual predictive check (pcVPC) were used to judge the final model (Bergstrand et al., 2011). The terminal elimination half-life (t_1/2_) was calculated as t_1/2_ = ln (2)/k_el_, where k_el_ is the individual empirical Bayes estimate of the ACV elimination constant.The software NONMEM was used to simulate different drug administration schedules based on the final POP/PK model. The simulation included dosages in the range 15–30 mg/kg administered as standard (i.e., 1 h), prolonged (i.e., 2 and 3 h) and continuous IV infusions every 6 and 8 h in 1,000 individuals for each dose level. C_min_ values higher than 0.56 or 1.156 mg/L in at least 50% of patients or C_max_ values > 25 mg/L in less than one-fourth of patients were considered as the desired pharmacokinetic targets in patients grouped according to the presence of augmented renal clearance (ARC) or not (i.e., eGFR >250 or ≤250 ml/min/1.73 m^2^, respectively) (Abdalla et al., 2020).

### Statistical Analysis

Data are presented as mean ± standard deviation (SD), median and minimum-maximum range, or 95% confidence interval (95% CI) according to the parameter described. Statistical computations (i.e., unpaired Student’s t-test with Welch’s correction, Mann-Whitney test, ANOVA, Fisher exact test) were performed using Prism 5.0 (GraphPad Software Inc., La Jolla, CA, United States) after checking for normal distribution of values (when appropriate) by the Kolmogorov-Smirnov test, and the significance level was set at *p* < 0.05.

## Results

### Patients and Acyclovir Monitoring

**FIGURE 1:**
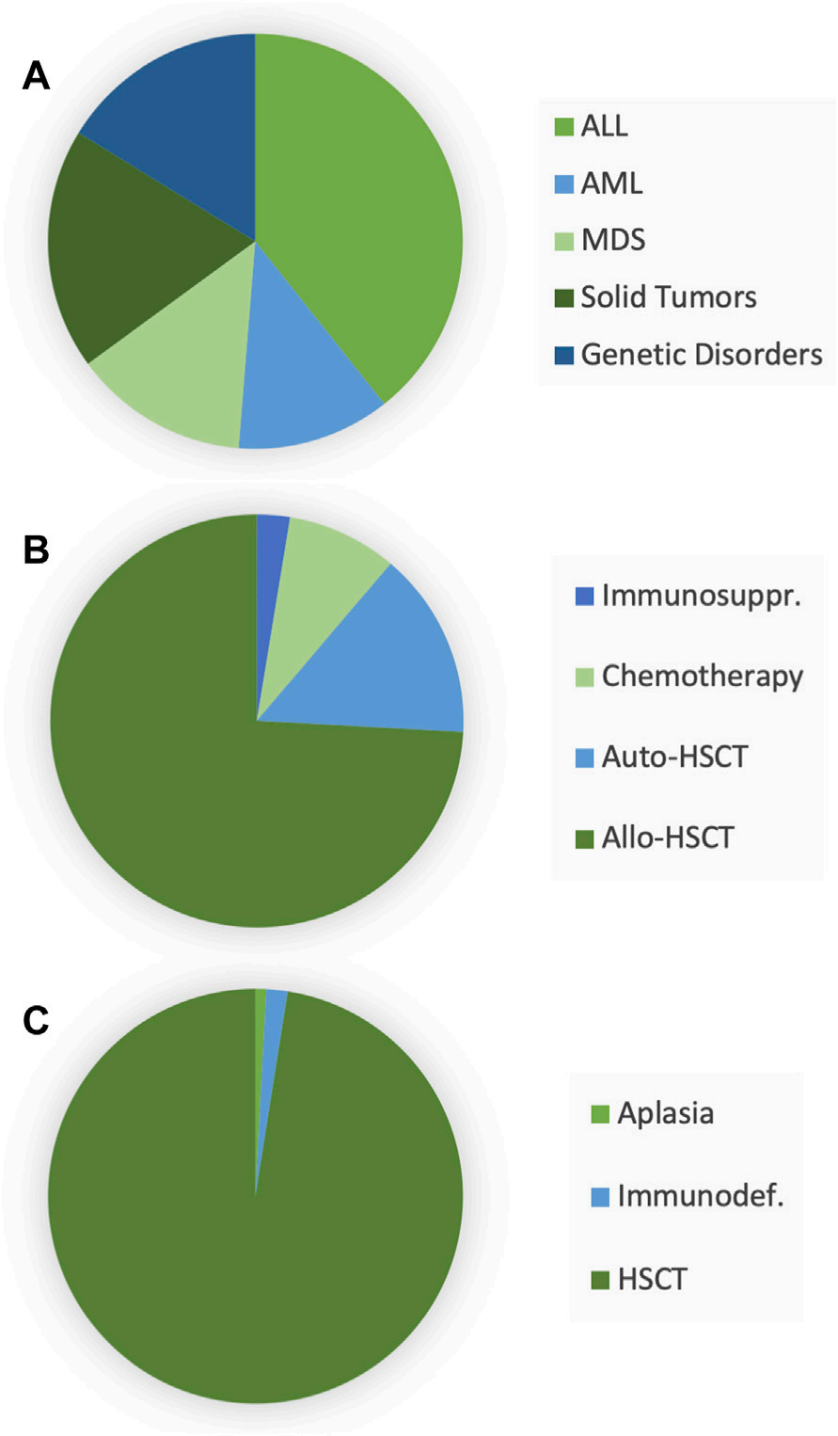
Characteristics of the 120 enrolled patients: initial diagnosis **(A)**, first-line therapy **(B)**, and reasons for acyclovir administration **(C)**. Abbreviations: ALL, acute lymphoblastic leukemia; AML, acute myeloblastic leukemia; MDS, myelodysplastic syndrome; HSCT, hematopoietic stem cell transplantation.

**FIGURE 2:**
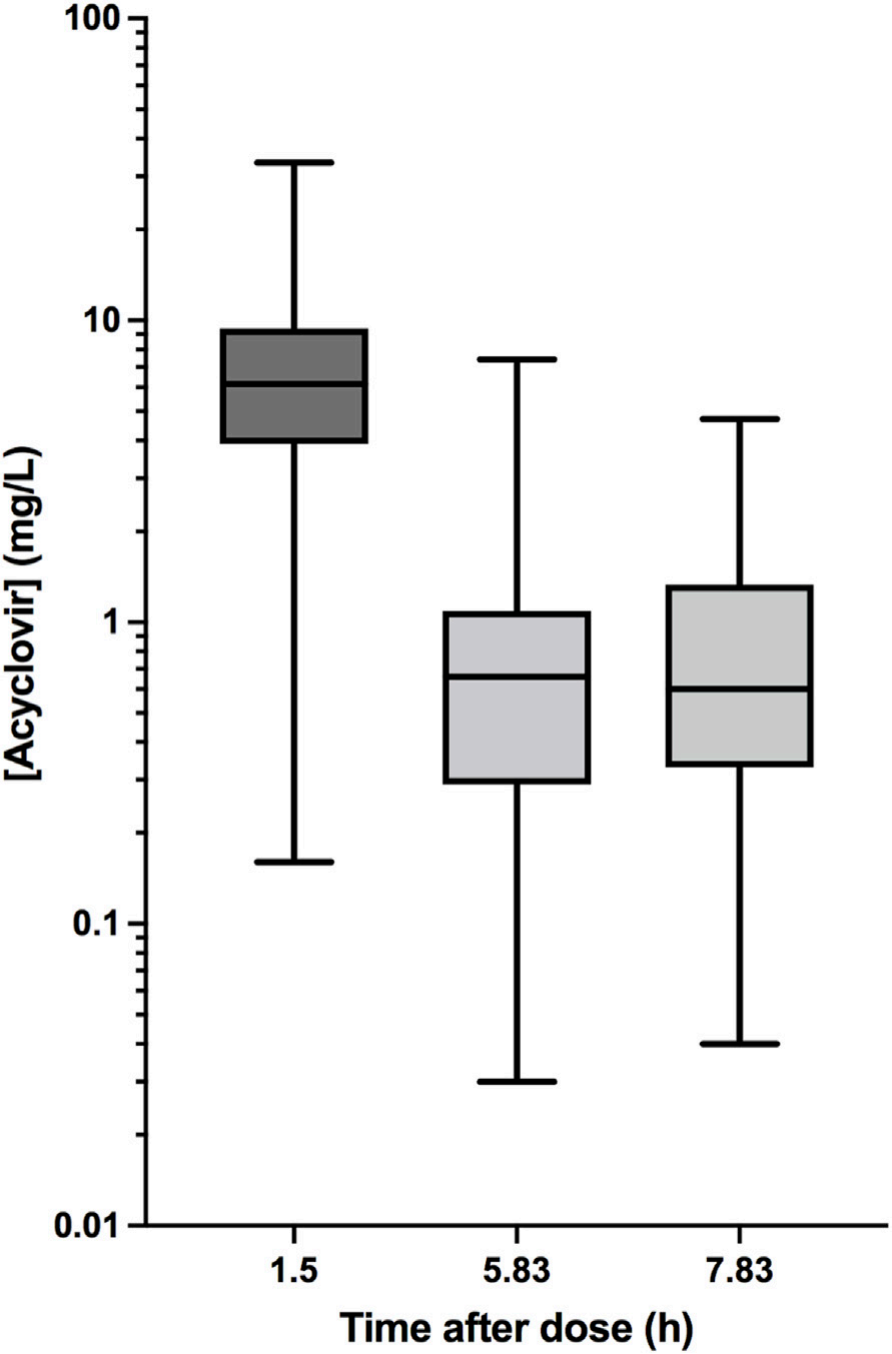
Box-and-whiskers plot of single measured plasma concentrations of acyclovir 30 min after the end of the infusion (1.5 h) and 10 min before the next dose in 6-h (5.83 h) and 8-h (7.83 h) regimens.

The current database included 73 boys and 47 girls (age, mean ± SD, 8.0 ± 5.2 and 9.5 ± 4.6 years, respectively; Table 1). Most patients were affected by hematological malignancies and addressed to allogenic HSCT (Figure 1). Twenty-six patients received ACV to treat a viral infection caused by HSV or VZV, whereas in the remaining 94 children the drug was administered for prophylaxis. Along with the treatment, blood samples were withdrawn to perform ACV monitoring of plasma concentrations in 120, 54, and 13 patients on a first, second, and third occasion, respectively. On the first occasion, measured C_max_ accounted for 7.6 ± 5.4 mg/L, while C_min_ values were 1.0 ± 1.1 and 0.85 ± 1.3 mg/L for the 6-h and 8-administration schedule of ACV, respectively, without significant differences across occasions (Figure 2). The dose was changed in 47 children (increased in 39) and 8 patients (increased in 7) at the second and third occasions, respectively.

**TABLE 1:** Characteristics of the 120 patients enrolled in the present study. There were not statistically significant differences between males and females.

	All patients (*n* = 120)	
	Mean ± SD	Median
Age (years)	8.6 ± 5.0	9.5
Weight (kg)	32.4 ± 19.1	27.8
Height (cm)	128.2 ± 30.0	131.5
Serum creatinine (mg/dl)	0.361 ± 0.206	0.320
eGFR (ml/min/1.73 m^2^)	228.1 ± 79.7	209.4
BSA (m^2^)	1.058 ± 0.426	0.998

eGFR, estimated glomerular filtration rate; BSA, body surface area.

In 26 patients (21.7%) who received ACV to treat herpetic infections, doses were 381.3 ± 199.2 mg (median, 400 mg) on the first occasion. In 11 and 4 patients, a second and a third occasions were available, with doses of 432.5 ± 150.9 mg (median 400 mg) and 500.0 ± 163.3 mg (median 500 mg), respectively. Among the measured ACV plasma concentrations, 15 and 11 patients had at least one C_min_ value > 0.56 and >1.156 mg/L, respectively. ACV administration was followed by a complete recovery, improvement, or infection control in approximately 75% of patients. In comparison, in the remaining individuals, the records showed a worsening of symptoms and signs (14.3% of patients) or the emergence of a further viral infection (i.e., cytomegalovirus, 9.5% of individuals). Measured C_min_ values in 3 out of 4 patients with poor response to therapy were greater than 1 mg/L, while in the fourth child, the C_min_ value accounted for 0.78 mg/L.

### POP/PK Modeling

A total of 187 pairs (120, 54, and 13 for the first, second, and third occasion, respectively) of ACV plasma concentrations for a total of 374 plasma concentration values were available for model development that started with a 1-compartment model with additive residual error (OFV, points 1,372.979). The residual variability was best described by a proportional error model (−141.269 points), while interindividual (IIV) and interoccasion variability (IOV) in systemic clearance (CL) significantly decreased the OFV value (−197.363 and −49.596 points, respectively). Furthermore, the introduction of IIV on the volume of distribution (V) ameliorated the model (−47.866 points), whereas IOV did not likely because changes in body weight across occasions were not significant (i.e., 32.2 ± 19.1, 30.9 ± 19.8, and 32.8 ± 22.7 kg, respectively at the first, second, and third occasion) and it was not considered in the following modeling.

**FIGURE 3:**
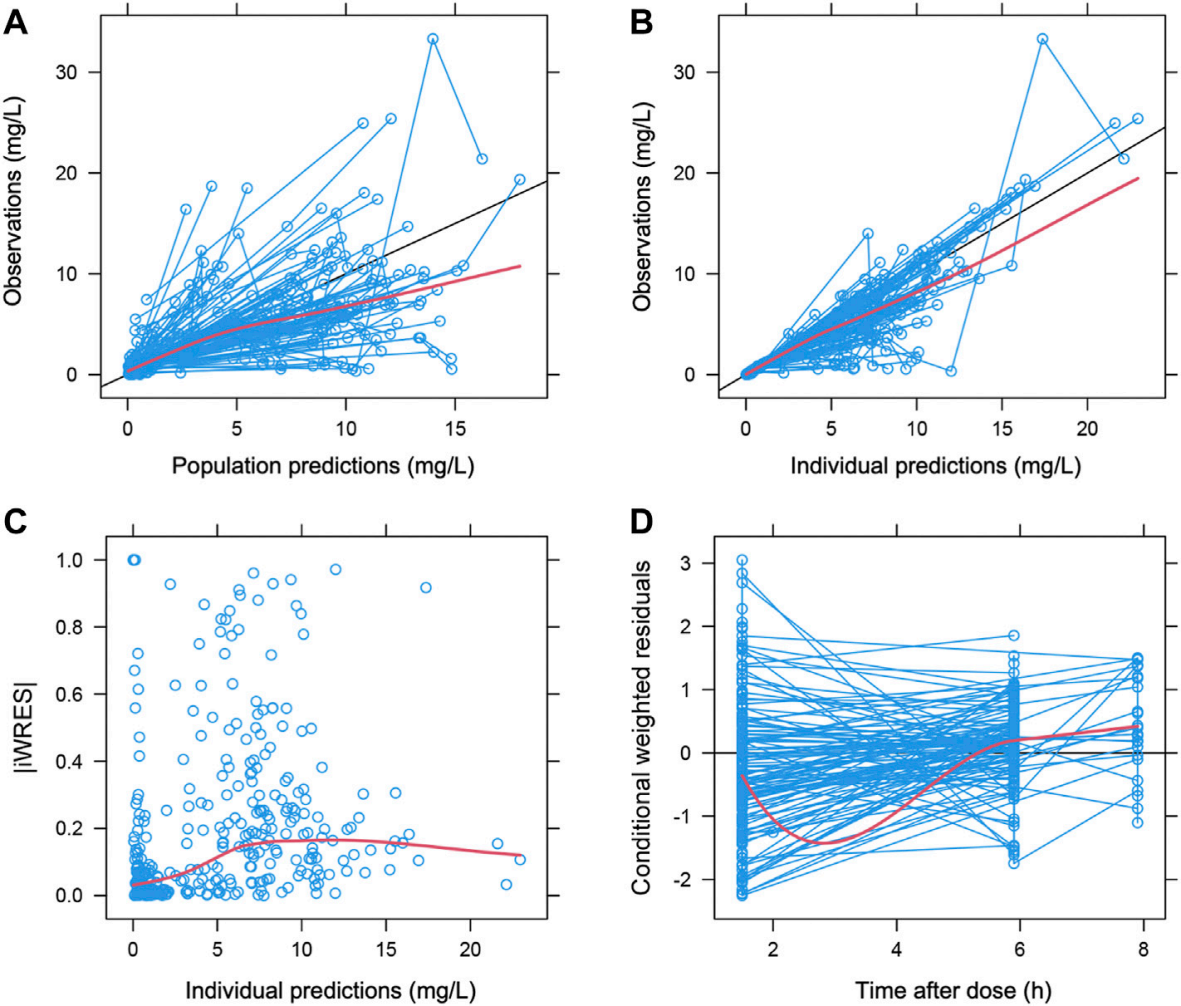
Goodness-of-fit plots. Population **(A)** and individual **(B)** prediction values vs. observations. **(C)**, absolute values of individual weighted residuals (iWRES) error vs. individual predictions and **(D)** conditional weighted residuals vs. time. Symbols, individual plasma concentrations of acyclovir. Red lines, regression, and Loess lines. Black line, line of identity.

**FIGURE 4:**
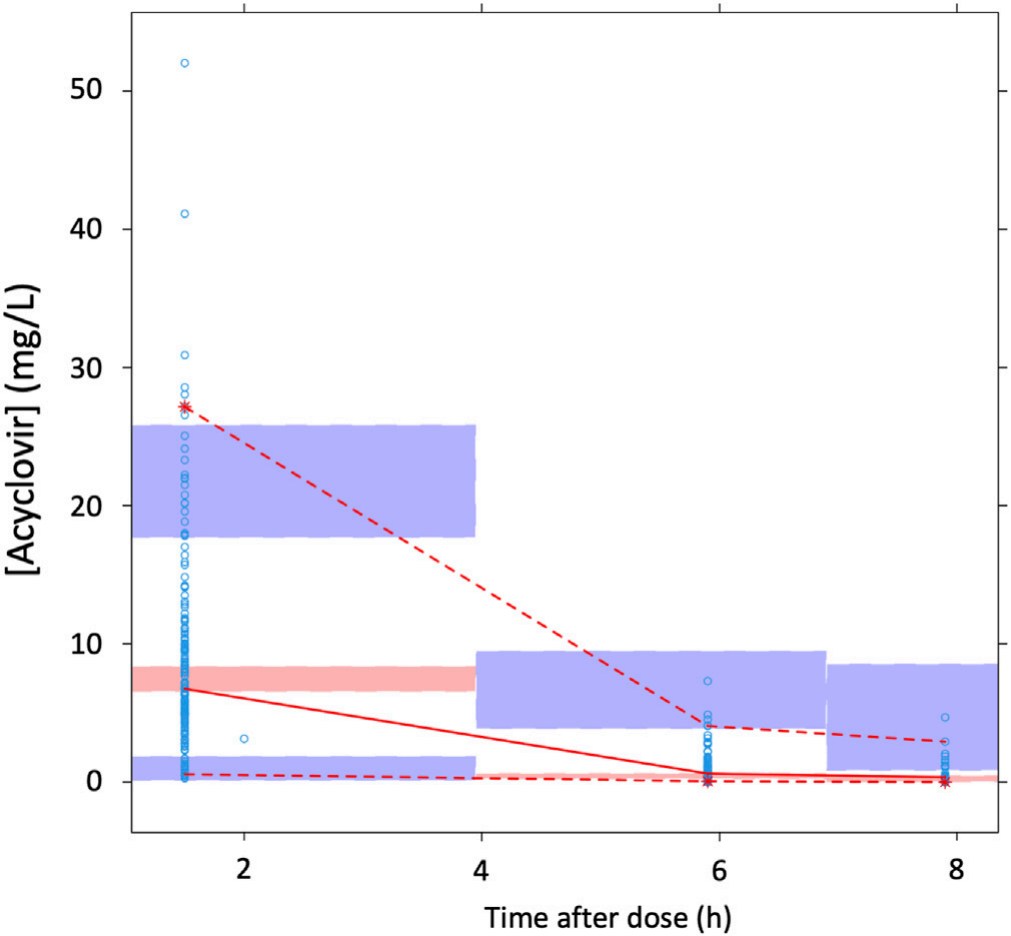
The prediction-corrected visual predictive check based on the present data. Open circles, individual plasma concentrations of acyclovir together with their median and 5th-95th percentiles (solid and dashed red lines, respectively). The 95% confidence intervals of the simulated median (pale pink) and 5th-95th percentiles (pale blue) are showed.

Among covariates with a possible effect on acyclovir pharmacokinetics, only eGFR on CL (−12.386 points) and body weight (with allometric scaling) on both CL and V (−22.811 and −12.664 points, respectively) did significantly decrease OFV; hence they were retained in the final model (Table 2). Among the GOF plots (Figure 3), the CWRES versus time after dose graph revealed an overprediction during the first few hours after the dose, which was likely dependent on the schedule of blood withdrawal, while the pcVPC plot did not entirely fit the lowest C_min_ and highest C_max_ values (Figure 4). However, the bootstrap analysis (with 1,000 resampled databases) resulted in good performance of the final model (Table 2), with nearly 90% of runs that ended successfully.

**TABLE 2:** Parameter values of the final model and bootstrap results obtained in 1,000 resampled databases.

	Final model		Bootstrap	
	Value	SE	Median	95% CI
OFV	889.024		873.384	756.091–994.564
CL (L/h)	6.184	1.630	6.186	2.965–9.985
V (L)	18.942	2.135	19.129	14.945–24.130
EGFR on CL	1.627	0.269	1.595	0.769–2.097
IIV_CL_	45.2%	25.4%	44.9%	23.9%–57.8%
IIV_V_	56.7%	33.8%	57.2%	13.0%–76.6%
IOV_CL_	20.0%	9.6%	19.9%	15.2%–31.5%
Residual variability	0.181	0.050	0.177	0.061–0.369
Shrinkage	η_1_ shrinkage	19.2	η_2_ shrinkage	24.7

Note: final model equations: CL (L/h) = [6.184 + (eGFR_i_/209.4)^1.627^ + (WGT/27.8)^0.75^] ^(η1 + IOV)^ and V (L) = [18.942 + (WGT/27.8)^1^] ^η2^.

SE, standard error; 95% CI, 95% confidence intervals; OFV, objective function value; CL, systemic clearance; V, volume of distribution; eGFR, estimated glomerular filtration rate; IIV, interindividual variability; IOV, interoccasion variability; WGT, body weight.

The analysis of PK parameters of the final model (showed in Table 3) did not demonstrate significant gender-based differences. The terminal t_1/2_ accounted for 1.364 ± 0.614 h, while IIV_CL_ and IOV_CL_ values were 46.4% and 20.0%, respectively.

**TABLE 3:** Values of the pharmacokinetic parameter obtained in the present 120 children by the final POP/PK model. There were not statistically significant differences between males and females.

	All patients (*n* = 120)	
	Mean ± SD	Median
Dose (mg)	334.0 ± 158.2	350.0
CL (L/h)	14.0 ± 5.5	14.6
AUC (hxmg/L)	26.0 ± 13.2	23.3
V (L)	25.4 ± 8.7	26.1
k_el_ (h^−1^)	0.574 ± 0.188	0.558
t_1/2_ (h)	1.364 ± 0.614	1.237

CL, systemic clearance; AUC, area under the time-concentration curve; V, volume of distribution; kel, constant of elimination; t1/2, terminal elimination half-life.

The present CL and V values for a typical individual do match those previously obtained (Abdalla et al., 2020), despite the higher eGFR values calculated in the present patients (median, 209.4 ml/min/1.73 m^2^) with respect to previous ones (164 ml/min/1.73 m^2^).

More interestingly, the analysis of PK parameters between the first and the second occasion did not reveal significant differences for any pharmacokinetic parameter considered (Table 4). In agreement with these results, an additional analysis of ACV PK in 13 patients did not show significant differences across the three occasions ( Table 5).

**TABLE 4:** Pharmacokinetics of ACV across two occasions. Values are presented as mean ± SD (median) values.

Occasion	Dose (mg)	CL (L/h)	AUC (hxmg/L)	V (L)	t_1/2_ (h)
1	334.0 ± 158.2	14.0 ± 5.5	26.1 ± 13.2	25.4 ± 8.7	1.364 ± 0.614
(73M + 47F)	(350.0)	(14.1)	(23.3)	(25.4)	(1.237)
*P*	0.213**	0.440*	0.755**	0.300*	0.924**
2	367.9 ± 167.7	14.7 ± 5.4	26.1 ± 12.4	27.0 ± 9.4	1.356 ± 0.516
(37M + 17F)	(350.0)	(14.6)	(23.9)	(28.0)	(1.238)

Note: *, Student’s t test; **, Mann-Whitney test.

CL, systemic clearance; AUC;area under the time-concentration curve; V, volume of distribution; t_1/2_, terminal elimination half-life.

**TABLE 5:** Pharmacokinetics of ACV across three occasions in 13 patients.

Occasion	Dose (mg)	CL (L/h)	AUC (hxmg/L)	V (L)	t_1/2_ (h)
1	199.2 ± 157.8 (123.0)	12.5 ± 5.3 (13.3)	17.4 ± 11.8 (13.1)	22.5 ± 10.9 (20.2)	1.310 ± 0.501 (1.100)
2	283.1 ± 188.6 (250.0)	11.74 ± 4.7 (12.5)	26.7 ± 21.4 (20.0)	24.4 ± 11.5 (25.5)	1.533 ± 0.816 (1.425)
3	298.5 ± 177.6 (300.0)	12.3 ± 4.0 (12.3)	24.3 ± 11.5 (24.4)	24.4 ± 11.5 (25.5)	1.441 ± 0.661 (1.319)
ANOVA	*0.500*	*0.989*	*0.832*	*0.618*	*0.321*

CL, systemic clearance; AUC, area under the time-concentration curve; V, volume of distribution; t_1/2_, terminal elimination half-life.

### POP/PK Simulation

The present simulation was based on the median values of covariates that were included in the final model. In particular, individual values of eGFR were randomly calculated from a distribution similar to that obtained from the present population of patients (i.e., mean and standard deviation values of 228.1 and 79.7 ml/min/1.73 m^2^, respectively), while body weight was fixed at 27.8 kg.

In agreement with a previous study (Abdalla et al., 2020), an ACV dose of 20 mg/kg every 6 h may ensure a C_min_ value > 0.56 and >1.125 mg/L in approximately 62.5% and 50.9% of the patients, respectively, while 28.6% of patients may experience a C_max_ value > 25 mg/L when the eGFR was ≤250 ml/min/1.73 m^2^ (Table 6). On the contrary, at eGFR values > 250 ml/min/1.73 m^2^, standard dosing regimens had a lower probability of target attainment, especially when the time interval between doses was 8 h (Table 6). The increase in dose to improve target attainment may expose the patients to an augmented risk of high peak plasma concentrations.

**TABLE 6:** Results from simulated regimens consisting in ACV dose ranging from 10 up to 30 mg/kg infused in 1 h (standard regimen). The percentages of patients who achieved C_min_ values > 0.56 and >1.125 mg/L or C_max_ values > 25 mg/L are showed according to an eGFR threshold of 250 ml/min/m^2^. Each regimen consisted of 1,000 simulated patients.

	1-h infusion	Time interval between doses: 6 h				
	Dose (mg/kg)	10	15	20	25	30
eGFR ≤ 250	C_min_ > 0.56	50.9	57.8	62.5	65.7	67.9
	C_min_ > 1.125	35.2	45.1	50.9	54.6	57.8
	C_max_ > 25	1.0	10.1	28.6	49.1	67.3
eGFR > 250	C_min_ > 0.56	37.8	44.8	50.6	54.0	59.0
	C_min_ > 1.125	22.2	32.4	37.9	42.5	44.8
	C_max_ > 25	0.6	5.7	21.3	36.5	55.6
		**Time interval between doses: 8 h**				
eGFR ≤ 250	C_min_ > 0.56	31.2	39.7	44.4	47.2	50.1
	C_min_ > 1.125	19.1	26.4	31.2	35.0	39.6
	C_max_ > 25	1.0	8.9	25.0	44.2	63.8
eGFR > 250	C_min_ > 0.56	20.1	27.1	32.8	35.7	37.6
	C_min_ > 1.125	11.1	15.6	20.1	24.8	26.8
	C_max_ > 25	0.6	5.4	20.6	34.9	52.4

eGFR, estimated glomerular filtration rate; C_min_, minimum plasma concentration; C_max_, maximum plasma concentration.

**FIGURE 5:**
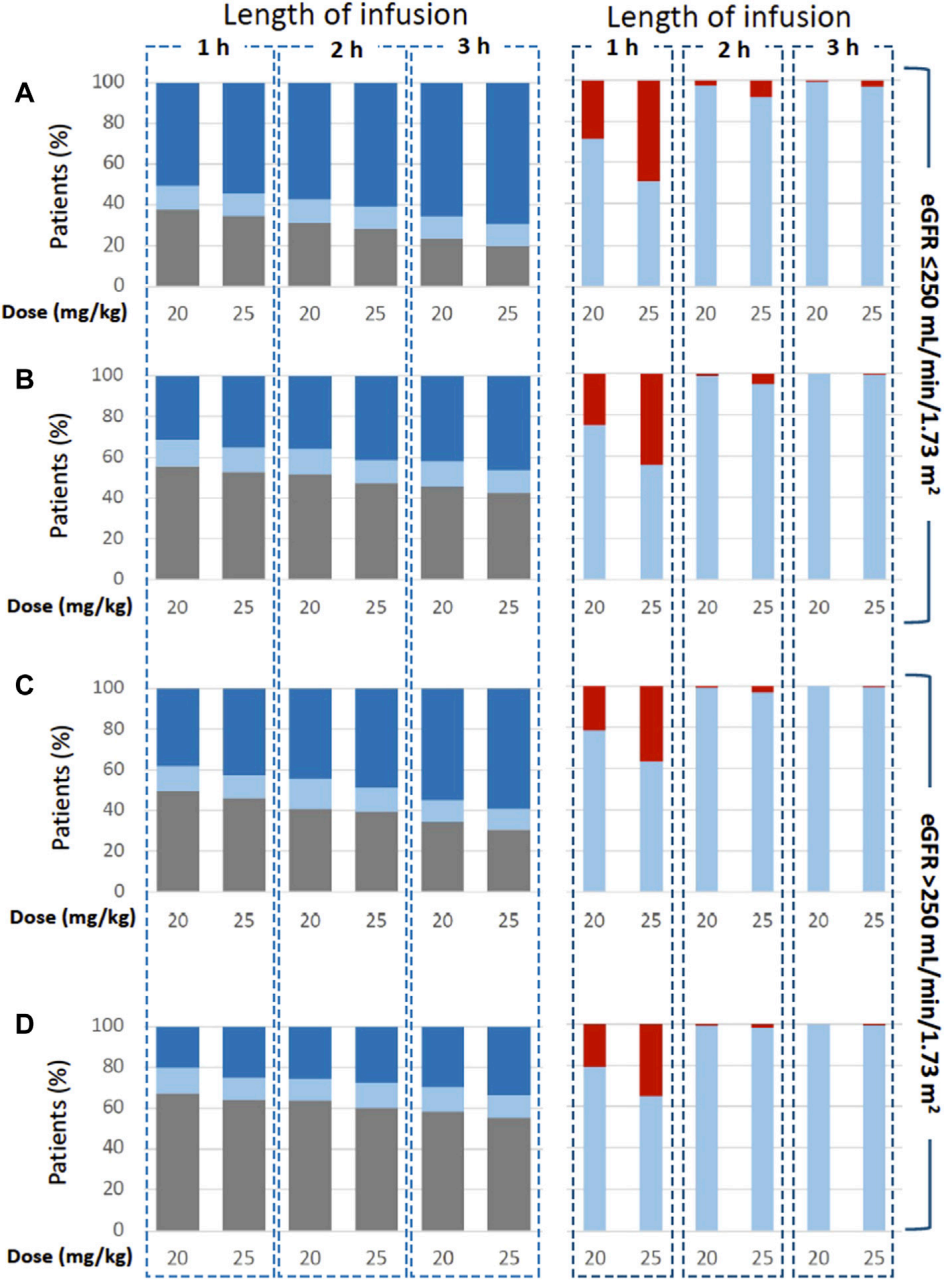
Simulated regimens consisting in ACV 20 or 25 mg/kg administered as 1-h, 2-h and 3-h infusion every 6 h [graphs **(A,C)**] and 8 h [graphs **(B,D)**]. Plots show the probability of target attainment (on the left) according to C_min_ values < 0.56 (grey), ≥0.56-<1.125 (pale blue) and >1.125 mg/L (blue). Moreover, graphs show the probability of achieving C_max_ values < 25 (pale blue) and >25 mg/L (red).

The following simulation of prolonged 2- and 3-h IV infusions resulted in an increased probability of ACV C_min_ values > 0.56 and >1.125 mg/L (Figure 5; Table 7). Of note, the reduced rate of infusion resulted in a lower probability of C_max_ values > 25 mg/L. In line with these results, simulated continuous infusions at doses of 10 mg/kg every 8 h were associated with C_min_ values > 0.56 mg/L in all patients regardless of the eGFR value. When the desired C_min_ threshold was set at 1.125 mg/L, 96.1% and 90.5% of patients achieved the target when the eGFR was ≤250 (C_min_ = 3.18 ± 1.69 mg/L) and >250 ml/min/1.73 m^2^ (C_min_ = 2.51 ± 1.26 mg/L), respectively.

**TABLE 7:** Results from simulated regimens consisting in ACV doses of 20 and 25 mg/kg infused in 1 h (standard regimen) or in prolonged infusions (2 and 3 h). The percentages of patients who achieved C_min_ values > 0.56 and >1.156 mg/L or C_max_ values > 25 mg/L are showed according to an eGFR threshold of 250 ml/min/m^2^. Each regimen consists of 1,000 simulated patients.

		Time interval between doses: 6 h					
		1-h infusion		2-h infusion		3-h infusion	
	Dose (mg/kg)	20	25	20	25	20	25
eGFR ≤ 250	C_min_ > 0.56	62.5	65.7	69.3	72.1	76.9	79.9
	C_min_ > 1.125	50.9	54.6	57.4	61.0	66.0	69.6
	C_max_ > 25	28.6	49.1	2.5	8.0	0.6	3.2
eGFR > 250	C_min_ > 0.56	50.6	54.0	59.2	60.5	65.6	69.4
	C_min_ > 1.125	37.9	42.5	44.3	48.7	54.8	58.9
	C_max_ > 25	21.3	36.5	0.6	2.9	0.0	0.3
		**Time interval between doses: 8 h**					
eGFR ≤ 250	C_min_ > 0.56	44.4	47.2	48.3	5 2.6	54.3	57.3
	C_min_ > 1.125	31.2	35.0	35.5	41.2	41.9	46.2
	C_max_ > 25	25.0	44.2	1.0	5.1	0	0.6
eGFR > 250	C_min_ > 0.56	32.8	35.7	36.3	39.9	41.8	44.7
	C_min_ > 1.125	20.1	24.8	25.5	27.7	29.6	33.6
	C_max_ > 25	20.6	34.9	0.6	1.6	0	0.3

eGFR, estimated glomerular filtration rate; C_min_, minimum plasma concentration; C_max_, maximum plasma concentration.

## Discussion

The present study was performed in a homogeneous population of oncologic paediatric patients receiving IV ACV for prophylaxis or to treat HSV and VZV infections. The final findings of the POP/PK analysis demonstrate a high variability between and within individuals that warrants the adoption of therapeutic drug monitoring. Furthermore, the simulation suggested that prolonged IV infusions could increase concentrations in the therapeutic range while reducing the risk of toxic peak concentrations.

ACV is a fundamental agent for prophylaxis and treatment of herpes virus infections, especially in HSCT patients or those who received high-dose antineoplastic chemotherapy like those enrolled in the present study. However, appropriate use of ACV depends on maintaining effectiveness through plasma concentrations above the threshold of sensitivity of known viral strains while reducing the risk of toxic concentrations identified in plasma levels >25 mg/L (Abdalla et al., 2020). The achievement of those goals may be severely influenced by patients’ characteristics, especially in children. Indeed, previous paediatric studies have clearly demonstrated that the variability in ACV pharmacokinetics is better explained by the renal function rather than dose (De Miranda and Blum, 1983), meaning that including plasma creatinine or eGFR within the model reduced IIV_CL_ and predicted the individual values with respect to observations (Zeng et al., 2009). In addition to this, the variability in both CL and V_d_ was significantly associated with body weight (Zeng et al., 2009; Abdalla et al., 2020). The present findings do confirm those conclusions in the largest population of enrolled patients published so far, showing a large interpatient and intrapatient variability that requires the adoption of TDM protocols. In the present study the unexplained IIV_CL_ (46.3%) was accompanied by a IOV_CL_ that accounted for 20.0%. Together, these values strengthen the benefit to include IOV in the estimation of individual PK parameters (Karlsson and Sheiner, 1993), and do sustain the monitoring of ACV concentrations during chemotherapy, because the time-varying covariates help to explain the variability across different occasions. Since it is possible that “the magnitude of IOV increases with the time between study occasions” (Karlsson and Sheiner, 1993), the mean/median interval time between two consecutive occasions in the present study was 16.5/8 days (minimum-maximum range, 2–165 days), likely explaining the lower IOV_CL_ with respect to IIV_CL_.

Some characteristics of the present study have to be pointed out. First of all, the number of enrolled patients did allow the analysis of patients who received IV ACV only, thus overcoming the variability associated with the oral administration of ACV or its prodrug valacyclovir. Second, the database used to develop the POP/PK model included plasma concentrations obtained at fixed time points that corresponded to those adopted in TDM protocols (Di Paolo et al., 2013) instead of dense blood sampling (Zeng et al., 2009), or random time points (Abdalla et al., 2020). The choice of the sampling scheme may depend on several factors, but ultimately the present POP/PK model returned PK estimates very similar to those published by other studies (Zeng et al., 2009; Abdalla et al., 2020). For example, the present mean values of CL (0.54 L/h/kg) and V (0.97 L/kg) were comparable to those previously reported in children with a mean bodyweight of 20 kg (Eksborg et al., 2002; Nadal et al., 2002). Analogous conclusions can be drawn for terminal t_1/2_ (De Miranda and Blum, 1983; Zeng et al., 2009) and the IOV_CL_ value (20.0%), which was in agreement with the value (19.2%) found in a previous study (Zeng et al., 2009). Even the simulation part of the present study showed a concordance with previous findings. For example, ACV doses of 10–20 mg/kg administered as a conventional 1-h IV infusion every 6 h showed almost identical percentages of toxic concentrations (Abdalla et al., 2020). Moreover, the present findings are suggesting that a 20-mg/kg dose every 6 h may achieve effective concentrations in approximately 50% of patients, while the dose should be increased in children with ARC.

The study’s novelty resides in the simulation of alternative regimens of acyclovir administration. With a short terminal half-life, the maintenance of effective C_min_ values is guaranteed by higher doses of acyclovir (i.e., 20 mg/kg) or more frequent dosing (i.e., every 6 h instead of 8 h). However, those high-dose-intensity regimens may result in high C_max_ values that could expose the patients to the risk of toxicities, while ineffective through concentrations may be measured especially in patients with ARC (Abdalla et al., 2020). As observed for other antimicrobial drugs that have a short plasma half-life, a prolonged (i.e., 2 or 3 h) or continuous infusion together with an appropriate dose increase may allow the achievement of effective target plasma concentrations. Indeed, the simulation of a prolonged infusion of a 20 mg/kg dose resulted in an increased percentage of patients achieving the predefined PK targets for the standard 1-h IV infusion. The percentage of patients who achieve effective C_min_ values sharply increased when the simulation considered a continuous infusion, even with a low dose-intensity regimen, consisting of 10-mg/kg doses every 8 h. Noteworthy, fragile patients affected by severe HSV infections were successfully cured with continuous IV infusions of acyclovir at higher doses (up to 30 mg/kg) (Engel et al., 1990; Kim et al., 2011). Interestingly, in most individuals “clinical response was seen within 72 h of continuous ACV administration” that “was well tolerated, even in patients with renal insufficiency.” Indeed, the authors did not observe any sign of toxicity, including hematological adverse reactions or deterioration in renal function.

Further studies have demonstrated that continuous infusions would prolong the time during which the ACV concentrations exceed the IC_50_ value for HSV and VZV and therefore may be considered a valid alternative to intermittent IV dosing (Spector et al., 1982; O'Leary et al., 2020). It is worth noting that in some cases, continuous ACV infusion was adopted to treat neonatal HSV encephalitis (Kakisaka et al., 2009; Cies et al., 2015; O’Leary et al., 2020). In particular, plasma concentrations were maintained above 3 mg/L (Cies et al., 2015) or even higher (5.5–8 mg/L) (O’Leary et al., 2020) to ensure cerebrospinal fluid concentrations of at last 1 mg/L.

Interestingly, a former study that enrolled 13 patients included 4 children who received continuous i.v., infusions of acyclovir at doses of 6.1–9.7 mg/kg/h from 7 up to 13 days (Fletcher et al., 1989). Plasma concentrations were ≥4.5 mg/L (up to 22.1 mg/L in some patients) and one child developed neutropenia, whereas none of the patients experienced renal insufficiency. In agreement with those observations, another study did not report signs or laboratory findings of systemic toxicity in 3 adolescents who received continuous i.v., infusions of ACV at doses of 7.2–28.8 mg/kg/day (Spector et al., 1982). Finally, the present simulation showed that a 10 mg/kg dose of ACV administered as a continuous infusion every 8 h allowed the achievement of therapeutic plasma concentrations in more than 90% of patients, regardless the eGFR value.

Those results do sustain the adoption of prolonged infusions (or continuous ones) because the reduced rate of drug infusion may be advantageous to decrease the risk of toxic C_max_ values. In particular, the percentage of patients at risk of higher peak plasma concentrations is abated, moving from a standard 1-h infusion to prolonged or continuous infusions. Additionally, a loading dose consisting of a 30-min infusion may precede the continuous infusion to ensure the achievement of therapeutic C_min_ values.

Although these premises of greater efficacy and good tolerability, the nephrotoxic effects of acyclovir in children have been associated with a variety of causes, as well as the concomitant administration of nephrotoxic drugs, a reduced eGFR at baseline, hypertension, older age, and obesity (Schreiber et al., 2008; Richelsen et al., 2018; Yalçınkaya et al., 2021). Therefore, these risk factors should be carefully evaluated, especially when dose-intense regimens were adopted (Fletcher et al., 1989).

In conclusion, the present findings confirm the high variability of ACV pharmacokinetics in immunocompromised children undergoing HSCT or myelotoxic chemotherapies, hence TDM protocols are recommended to adjust drug dosing. Indeed, standard dosing regimens seems adequate to achieve effective plasma concentrations of ACV, despite the drug could be ineffective in a variable percentage of some patients. The further increase in dosage may expose patients to an augmented risk of toxicities. Noteworthy, alternative regimens based on prolonged IV infusions (i.e., 20 mg/kg as a 3-h infusion every 6 h) or even continuous infusions (i.e., 10 mg/kg every 8 h) may increase the efficacy of ACV while reducing the risk of highest plasma peaks and sparing patients from severe toxicities. Prospective trials adopting TDM protocols are required to confirm the present findings.

## Data Availability

The original contributions presented in the study are included in the article/Supplementary Material, further inquiries can be directed to the corresponding author.

## Footnotes

1 ICON plc. NONMEM^®^. [Accessed 1 September 2021]. https://www.iconplc.com/innovation/nonmem/.
